# Ultrastructural insights into pathogen clearance by autophagy

**DOI:** 10.1111/tra.12723

**Published:** 2020-03-04

**Authors:** Chieko Kishi‐Itakura, Nicholas T. Ktistakis, Folma Buss

**Affiliations:** ^1^ Cambridge Institute for Medical Research, Keith Peters Building University of Cambridge Cambridge UK; ^2^ Signalling Programme Babraham Institute Cambridge UK

**Keywords:** autophagy, electron microscopy, phagophore, *Salmonella*, xenophagy

## Abstract

Autophagy defends cells against proliferation of bacteria such as *Salmonella* in the cytosol. After escape from a damaged *Salmonella*‐containing vacuole (SCV) exposing luminal glycans that bind to Galectin‐8, the host cell ubiquitination machinery deposits a dense layer of ubiquitin around the cytosolic bacteria. The nature and spatial distribution of this ubiquitin coat in relation to other autophagy‐related membranes are unknown. Using transmission electron microscopy, we determined the exact localisation of ubiquitin, the ruptured SCV membrane and phagophores around cytosolic *Salmonella*. Ubiquitin was not predominantly present on the *Salmonella* surface, but enriched on the fragmented SCV. Cytosolic bacteria without SCVs were less efficiently targeted by phagophores. Single bacteria were contained in single phagophores but multiple bacteria could be within large autophagic vacuoles reaching 30 μm in circumference. These large phagophores followed the contour of the engulfed bacteria, they were frequently in close association with endoplasmic reticulum membranes and, within them, remnants of the SCV were seen associated with each engulfed particle. Our data suggest that the *Salmonella* SCV has a major role in the formation of autophagic phagophores and highlight evolutionary conserved parallel mechanisms between xenophagy and mitophagy with the fragmented SCV and the damaged outer mitochondrial membrane serving similar functions.

## Graphical abstract

Xenophagy is a specialised selective autophagy pathway for the cellular defence against pathogens. The conjugation of ubiquitin to invasive bacteria is an important step in xenophagy, however, the spatial distribution of the ubiquitin coat remains unclear. Here, we used electron microscopy to analyse the topology of the *Salmonella*‐host membrane system and the distribution of ubiquitin. Our results demonstrate that proteins of the ruptured *Salmonella*‐containing vacuole rather than bacterial surface proteins are the major ubiquitination target.

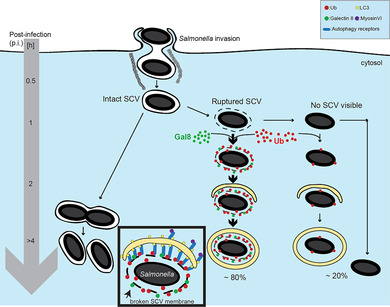

## 1 INTRODUCTION

Autophagy is a catabolic pathway to maintain cellular homeostasis by generating nutrients under starvation conditions or by eliminating damaged organelles and dysfunctional cellular components as a quality control mechanism.^1^, ^2^ This pathway is also important for the innate immunity response to infection by targeting intracellular bacteria residing in phagosomes, damaged vacuoles and the cytosol.^3^, ^4^ The defence process to restrict and eliminate cytosolic bacteria is activated through several parallel events.^5^, ^6^, ^7^ A recently discovered pathway of direct targeting of intracellular pathogens inside the vacuole is through recruitment of ATG16L1 by the vacuolar‐ATPase onto the bacteria‐surrounding membrane, which is blocked by the bacterial effector SopF.^8^ In addition, an early “eat‐me” signal in the form of lectins can be found on the inside of the ruptured SCV and detected by Galectin‐8 as early as 1 hour after infection.^9^ This Galectin‐8 recognised signal coincides with formation of a ubiquitin coat surrounding *Salmonella* at early time points. At later time points between 4 and 8 hours after infection, a second longer‐lasting ubiquitin signal appears in closer vicinity to the *Salmonella* surface if the bacteria are still not cleared by autophagy.^10^ The bacterial‐associated ubiquitin signal is recognised by selective autophagy receptors such as sequestosome 1 (SQSTM1/p62),^11^ Tax1 binding protein 1 (TAX1BP1/ CALCOCO3)^12^ and its paralogue nuclear domain 10 protein 52 (NDP52/CALCOCO2)^13^ as well as optineurin (OPTN).^14^ These selective autophagy receptors characteristically can bind to both ubiquitin on *Salmonella* and the LC3‐positive autophagic membranes, thereby enabling assembly of the phagophore. In addition to the LC3‐adaptor interaction, the way by which adaptors co‐operate with the rest of the autophagic machinery to recognise and engulf *Salmonella* is an area of active investigation. In general terms, it appears that early autophagy proteins such as members of the ULK complex, the phosphatidylinositol 3‐phosphate (PI3P) effectors WIPI proteins and the lipidation machinery component ATG16 all can recognise parts of the adaptor proteins. For example, it was recently shown that NDP52 forms a complex with FIP200 and SINTBAD/NAP1 leading to the recruitment of the autophagy machinery to *Salmonella* in the cytosol. The ULK complex localises to the Galectin‐8‐positive *Salmonella* surface, highlighting the importance of the damaged SCV for phagophore formation.^15^, ^16^ In addition, the WIPI2 PI3P effector promotes the localization of the TBK1 kinase to the invading *Salmonella* prior to autophagic engulfment (https://www.ncbi.nlm.nih.gov/ pubmed/27370208).

*Salmonella* first enters the cell interior through macropinocytosis and resides within a novel single membrane compartment termed SCV.^17^ The SCV gradually acquires characteristics of the endocytic compartment as it matures. Within the SCV, *Salmonella* can replicate or, alternatively, it can escape into the cytosol where it is found either “naked” or still partially associated with ruptured SCVs. Autophagy appears to be triggered by bacteria in ruptured SCVs as an elimination mechanism although it also has been suggested to enable repair of the damaged membranes.^18^, ^19^, ^20^ The sequence of ubiquitin‐triggered and autophagy receptor‐dependent recruitment of autophagosome membranes is well established, but the site of ubiquitination that initiates this sequence of events is less well‐known. Equally perplexing are the topological relationships between the bacterial outer membrane, the SCV and the phagophore double‐membrane as it is being formed through the action of adaptors and the autophagy core machinery. Finally, the stoichiometry of bacteria within the autophagic membranes is not entirely settled.

To establish the exact distribution of ubiquitin, autophagy receptors and LC3‐positive membranes, we have performed a detailed ultrastructural analysis of the *Salmonella*‐host membrane system. We show the distribution of ubiquitin, selective autophagy receptors and autophagosomal membranes using electron microscopy (EM). Our results demonstrate the exact topology of host and *Salmonella* membranes, ubiquitinated target proteins and components of the autophagy machinery and provide evidence that SCV membrane proteins are ubiquitination targets that are recognised by selective autophagy receptors, such as TAX1BP1 leading to the assembly of phagophores. Bacteria that have lost all remnants of SCV membranes are less likely to recruit autophagosomal membranes. These findings highlight the similarity between mitophagy and xenophagy; in the former, proteins associated with the outer mitochondrial membrane, which is reminiscent of the SCV, are ubiquitinated to promote clearance of mitochondria via autophagy.

## 2 RESULTS

### 2.1 Ultrastructural analysis of mouse fibroblasts after *Salmonella* infection reveals that several bacteria can be captured inside a single phagophore

**Figure 1:**
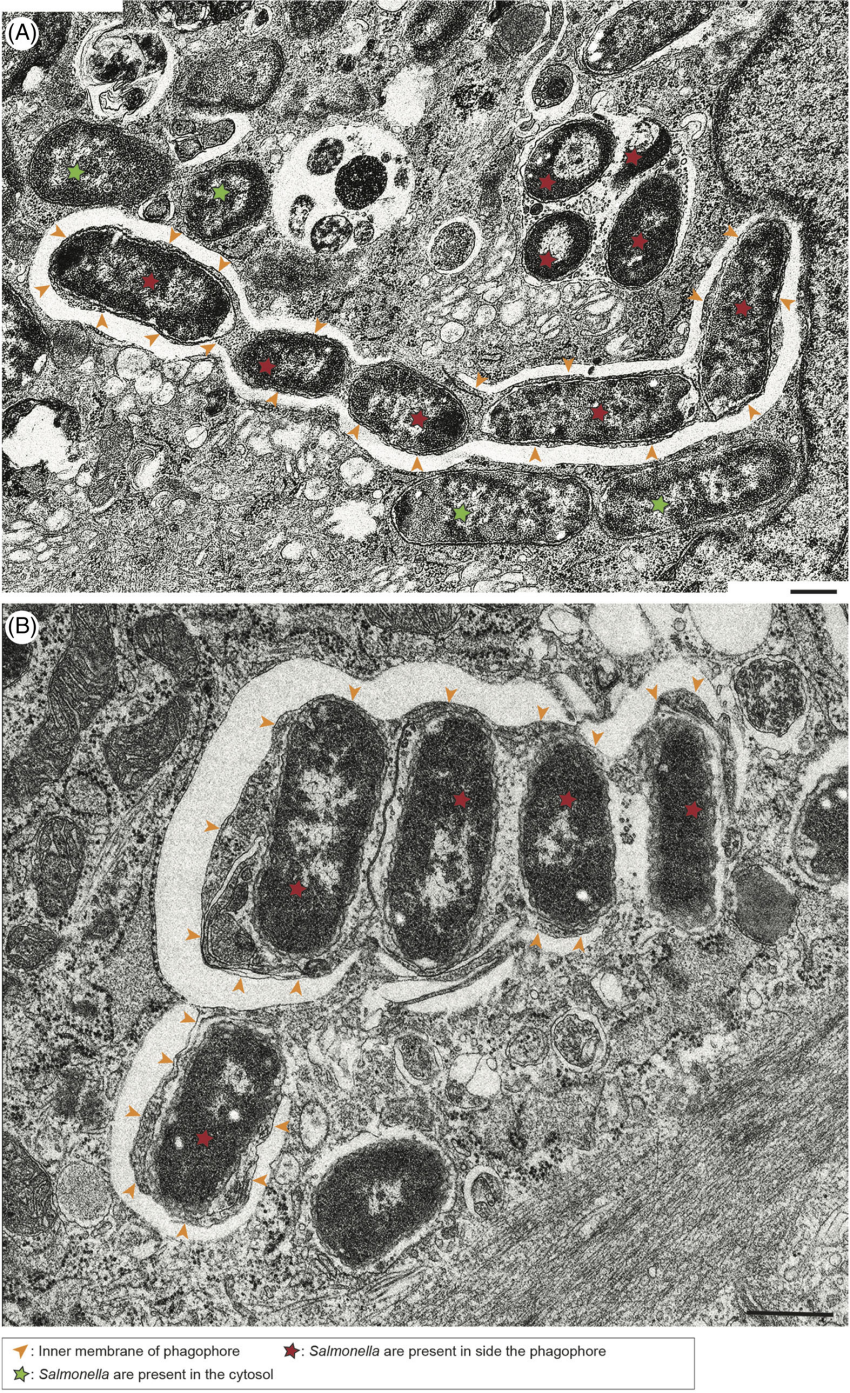
*Several Salmonellae* are surrounded by multiple phagophores, which fuse to form a single autophagosome. Transmission electron microscopy of mouse embryonic fibroblasts (MEF) cells infected with *Salmonella*‐Cherry for 4 h (A, B). *Salmonellae* inside the phagophore are highlighted by red stars and *Salmonellae* in the cytosol are marked with green stars. The orange arrowheads highlight the inner phagophore membranes. Scale bar, 500 nm

**Figure 2:**
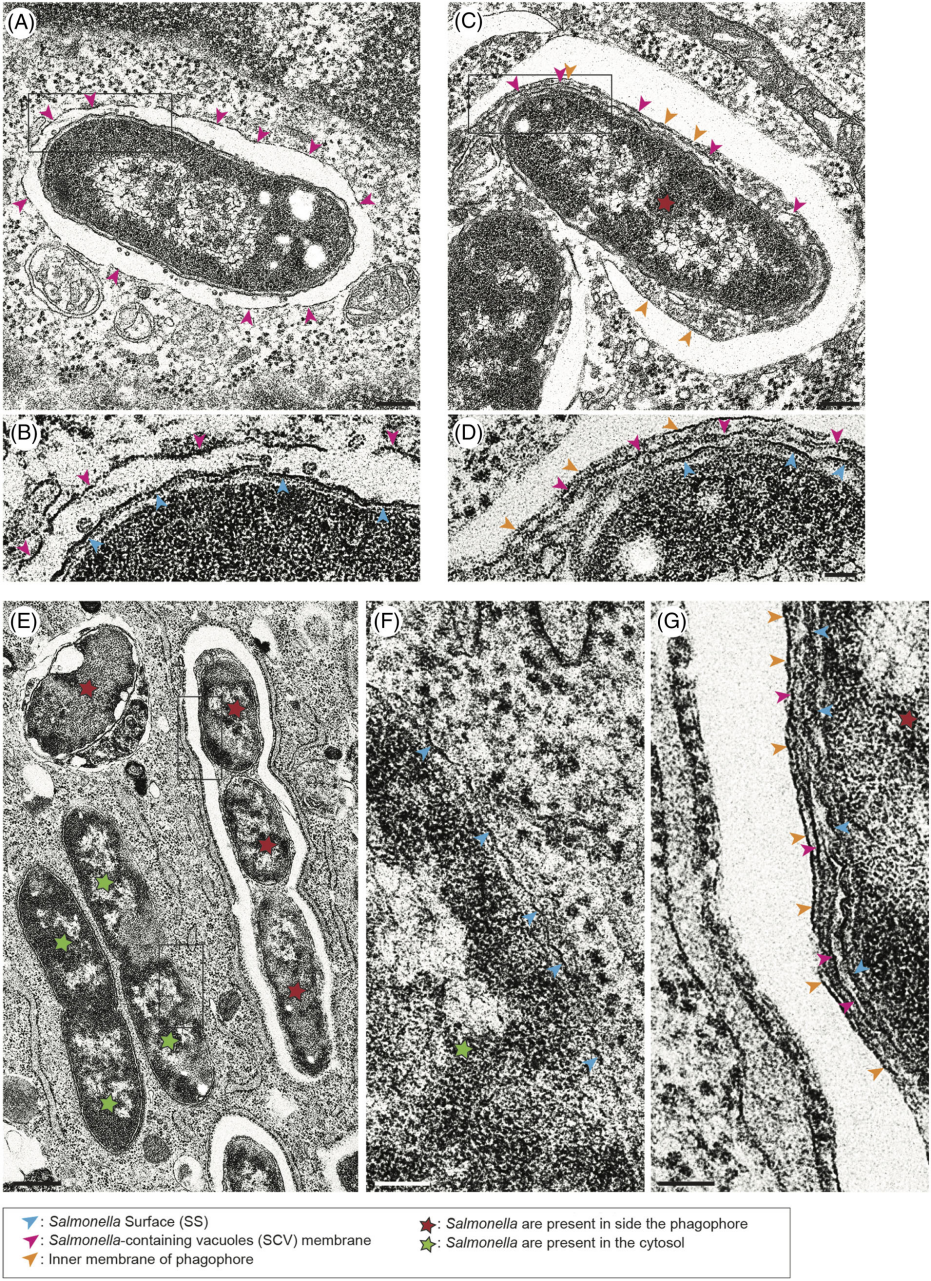
Fragments of the *Salmonella*‐containing vacuole (SCV) membrane are present between the *Salmonella* surface and the phagophore. Conventional electron microscopy of mouse embryonic fibroblasts (MEFs) infected with *Salmonella*‐Cherry for 1 h (A‐D) or 4 h (E‐G). (A) *Salmonella* resides in an SCV marked with pink arrowheads. Rectangle in (A) shows area enlarged in (B) with blue arrowheads pointing at the *Salmonella* surface (SS) and pink arrowheads at the SCV membrane. (C and D) Example of a single *Salmonella* surrounded by a growing phagophore. The phagophore membrane is highlighted by orange arrowheads, the SCV membrane by pink arrowheads and the *Salmonella* surface by blue arrowheads. Rectangle in (C) shows area enlarged in (D). Scale bars, 200 nm (panel A and C) and 50 nm (panel B and D). (E) High‐resolution electron microscopy image of membranes surrounding *Salmonella* inside the cytosol (green stars) or inside a single phagophore (red stars). Rectangles in (E) show area associated with *Salmonella* inside the cytosol (F, green star) or area of phagophore membrane (G, red star). The *Salmonella* surface membrane is labelled by blue arrowheads, the SCV membrane by pink arrowheads and the inner phagophore membrane by orange arrowheads. Scale bars, 500 nm (E), 100 nm (F and G)

To visualise and follow the distribution of invading bacteria at the ultrastructural level, mouse embryonic fibroblasts were incubated with wildtype *Salmonella* and processed for conventional transmission electron microscopy (TEM) 1, 2 or 4 hours after infection. On the resulting electron micrographs, bacteria are present 1‐hour post‐infection (p.i.) within the SCV (Figure 2A), in the cytosol without any obvious surrounding membranes (marked with a green star in Figure 1A and 2E) or enclosed fully or partially by phagophores (marked with a red star in Figure 1A and B and Figure 2C and E). Interestingly, phagophores not only capture single bacteria, but frequently we observed several *Salmonellae* engulfed by a single large phagophore, which appears to be zippered along the outline of the bacteria (Figure 1A and B and Figure 2E). In places several single phagophores closely align along neighbouring *Salmonellae*, suggesting that the large phagophores may originate through fusion of several smaller phagophore membranes (Figure 1B).

### 2.2 Ruptured SCV membranes are present between the *Salmonella* surface and the phagophore

We next used EM to perform a detailed analysis of the *Salmonella*‐host membrane topology. The bacterium itself is surrounded by a double membrane, an outer and an inner membrane separated by periplasm. The detailed topology of the *Salmonella* surface is shown in Figure S1A.

After invasion, the pathogen resides in the SCV, which allows replication of the bacteria. At the ultrastructural level, the SCV appears as a continuous single membrane often containing numerous very small vesicles in the space between the *Salmonella* surface (SS) and the SCV membrane (Figure 2A,B). SCV damage exposes the bacteria to the cytosol, which triggers the formation of a dense ubiquitin coat and the assembly of the phagophore. We next used conventional EM to visualise ultrastructural detail of phagophores on the bacterial surface, and found that fragments of the SCV membrane are often present between the *Salmonella* surface and the phagophore (Figure 2C,D,E and G). In contrast, “naked” cytosolic bacteria without an SCV membrane (green stars) are not surrounded by a phagophore (Figure 2E and F).

### 2.3 Galectin‐8‐positive SCV membranes colocalise with phagophores

**Figure 3:**
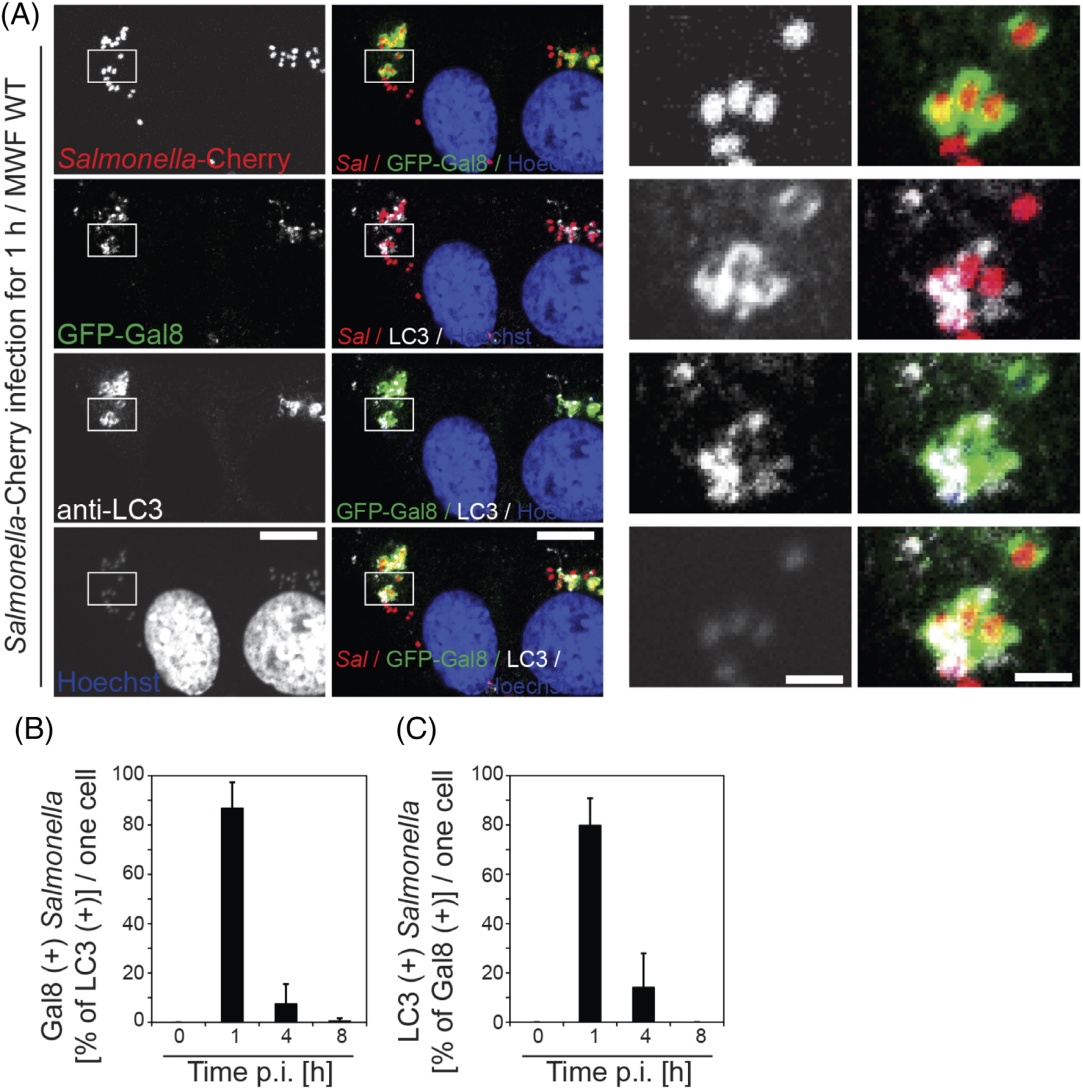
The majority of Galectin‐8‐positive *Salmonellae* are also LC3‐positive. (A) Mouse embryonic fibroblasts (MEF) cells transiently expressing GFP‐Galectin‐8 were infected with *Salmonella*‐Cherry for 1 h, processed for confocal microscopy and labelled for LC3 (far red). Nuclei were labelled with Hoechst (blue). Rectangles are shown enlarged in panels on the right side. Scale bars, 10 μm (left panels), 2 μm (right panels). (B) Quantification of the percentage of Galectin‐8‐ positive *Salmonellae* that are also LC3 positive and (C) the percentage of LC3‐positive *Salmonellae* that are also Galectin‐8 positive

Our next step was to determine whether SCV membranes are still present on cytosolic *Salmonellae* that are targeted by the autophagy machinery and assemble LC3‐positive membranes. To label broken SCV membranes GFP‐Galectin‐8 was transiently expressed in MEFs, which were then infected for 1 hour with Cherry‐*Salmonella*. In order to visualise autophagosomal membranes, the cells were also labelled with anti‐LC3 antibodies. We quantified the number of bacteria that are positive for Galectin‐8 and for LC3 and plotted the results as the percentage of LC3‐positive bacteria that also contain Galectin‐8 (Figure 3B) or the percentage of Galectin‐8‐positive bacteria that are surrounded by LC3‐positive membranes (Figure 3C). Our data show that more than 80% of *Salmonellae* that are positive for LC3 also contain the SCV marker Galectin‐8. These quantitative results support our ultrastructural observations that the formation of a phagophore coincides with the presence of SCV fragments on the *Salmonella* surface.

**Figure 4:**
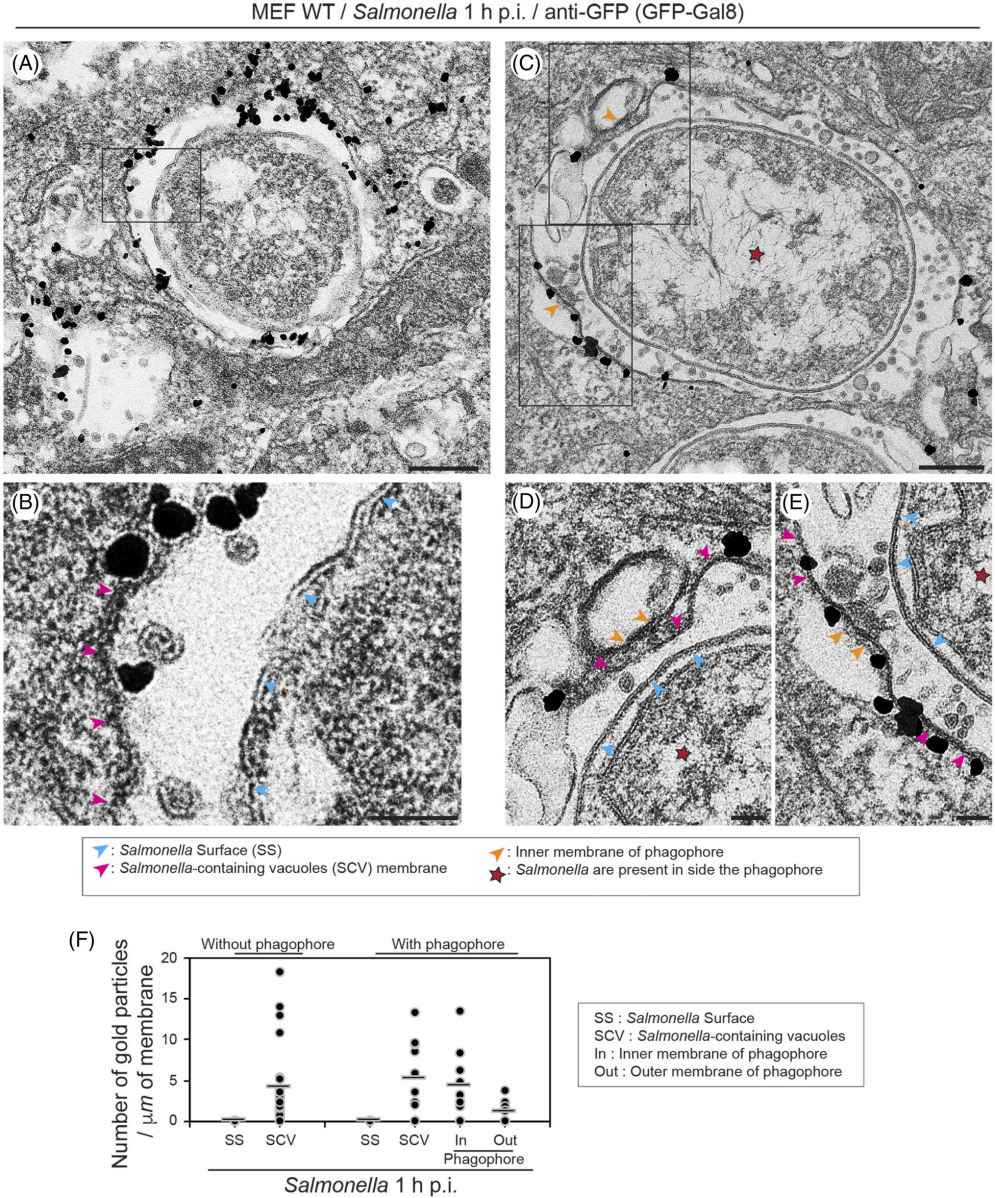
Galectin‐8‐positive *Salmonella*‐containing vacuole (SCV) fragments are between the *Salmonella* surface and the phagophore. (A) (C) Mouse embryonic fibroblasts (MEF) cells transiently expressing GFP‐Galectin‐8 were infected with *Salmonella*‐Cherry for 1 h, processed for immunoelectron microscopy and stained with anti‐GFP antibodies to detect Galectin‐8 distribution. Rectangle in A and C are enlarged in (B), (D) and (E). Pink arrowheads highlight position of SCV membrane, blue arrowheads the *Salmonella* surface and orange arrowheads the phagophore membrane. Scale bars, 200 nm (A and C), 50 nm (B, D and E). (F) Quantification of the number of gold particles per μm of membrane highlighting the distribution of GFP‐Galectin‐8 on the *Salmonella* surface (SS), the SCV, on the inside or the outside of the phagophore. Bars show average of each column

To further analyse whether the formation of a phagophore around cytosolic bacteria overlaps with the presence of Galectin‐8‐positive SCV membrane fragments, MEFs which transiently express GFP‐Galectin‐8 were infected with *Salmonella*‐Cherry for 1 hour and processed for immunoelectron micros to label the localization of Galectin‐8 with anti‐GFP. Strong labelling of Galectin‐8 on the membrane of SCV was detected (Figure 4A,B). Interestingly, Galectin‐8‐positive membranes appear to mark the sites where phagophores are forming on the bacterial surface (see Figure 4C‐E; orange arrows mark the inner membrane of the phagophore). Quantitation of the Galectin‐8 distribution further confirms the association of autophagosomal membranes with SCV membranes, which were identified by the presence of Galectin‐8 (Figure 4F). We also observed in the space between the *Salmonella* surface and the SCV membrane very small luminal vesicles typically found inside the vacuolar compartment surrounding the *Salmonella* (Figure 4A‐E).

**Figure 5:**
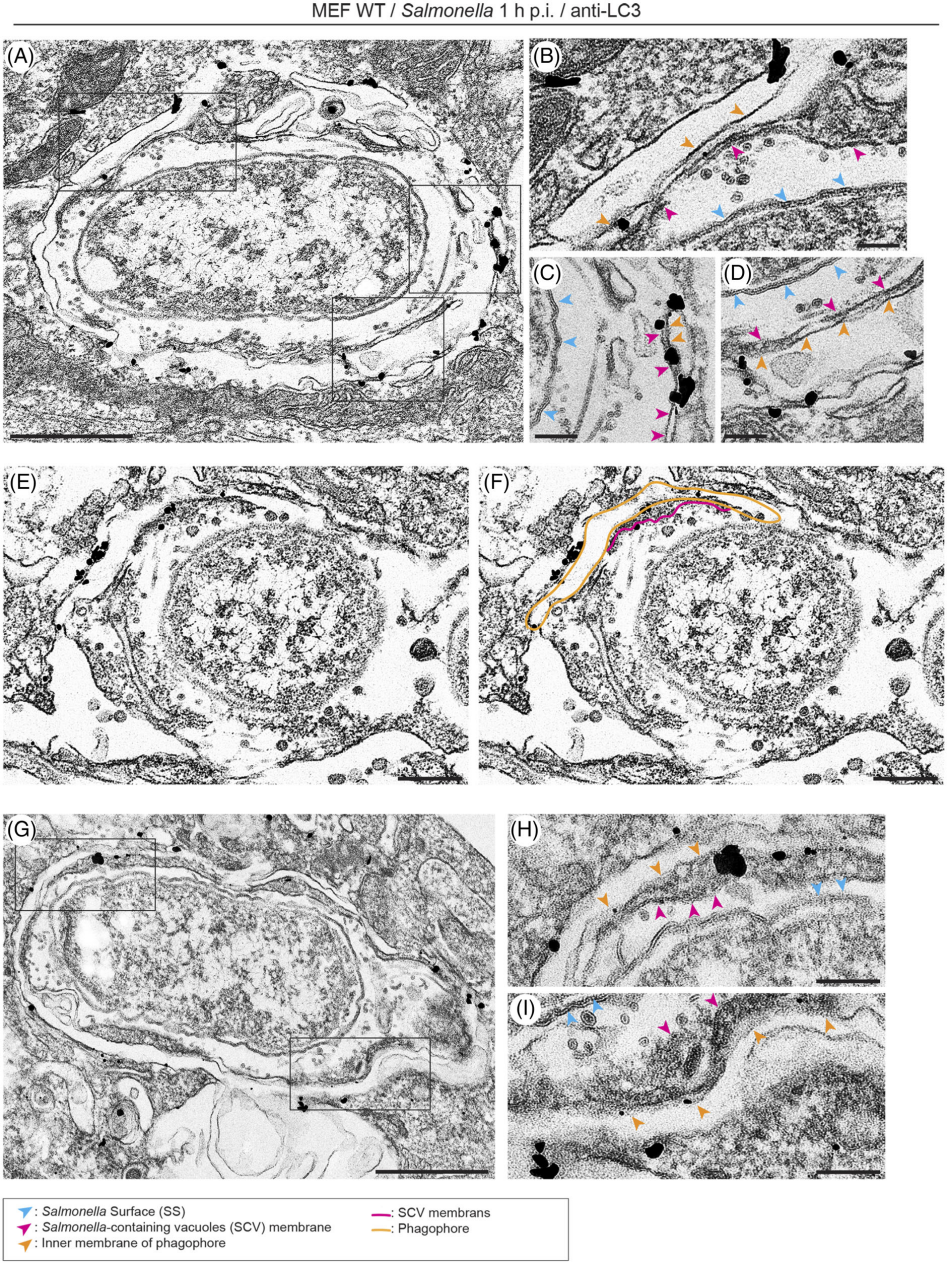
*Salmonella*‐containing vacuole (SCV) membranes are present where LC3‐positive phagophores are assembled. Mouse embryonic fibroblasts (MEF) cells were infected for 1 h with *Salmonella*, processed for immunoelectron microscopy and stained with anti‐LC3 antibodies. (A and G) Rectangles are shown enlarged in B, C, D, H and I. Scale bars, 500 nm (panel A and G), 100 nm (B, C, D, H and I). (E and F) LC3‐positive double membrane is coloured in orange and fragmented SCV membranes surrounding *Salmonella* are highlighted in pink in panel F. Scale bars, 200 nm (E and F)

To confirm the nature of the phagophore, MEF cells were infected for 1 hour with *Salmonella*‐Cherry, processed for immunoelectron microscopy and stained with anti‐LC3 antibodies (Figure 5). Formation of LC3‐positive double membranes surrounding a bacterium was observed outside a fragmented SCV, which still contains a large number of small internal vesicles (Figure 5A‐D and G‐I). In Figure 5E,F the LC3‐positive double membrane of the phagophore is coloured in orange and the fragmented SCV membranes surrounding *Salmonella* are highlighted in pink.

Thus, using immunoEM, we have been able to identify Galectin‐8‐positive SCV membranes at the site of phagophore formation (Figure 4D,E). Furthermore, in areas with very small luminal vesicles, which are contained inside the vacuolar compartment by the presence of an SCV membrane, we were able to observe the formation of LC3‐positive phagophores (Figure 5A‐I).

### 2.4 Galectin‐8‐positive SCVs are ubiquitin positive and recruit the autophagy receptors TAX1BP1 and p62

**Figure 6:**
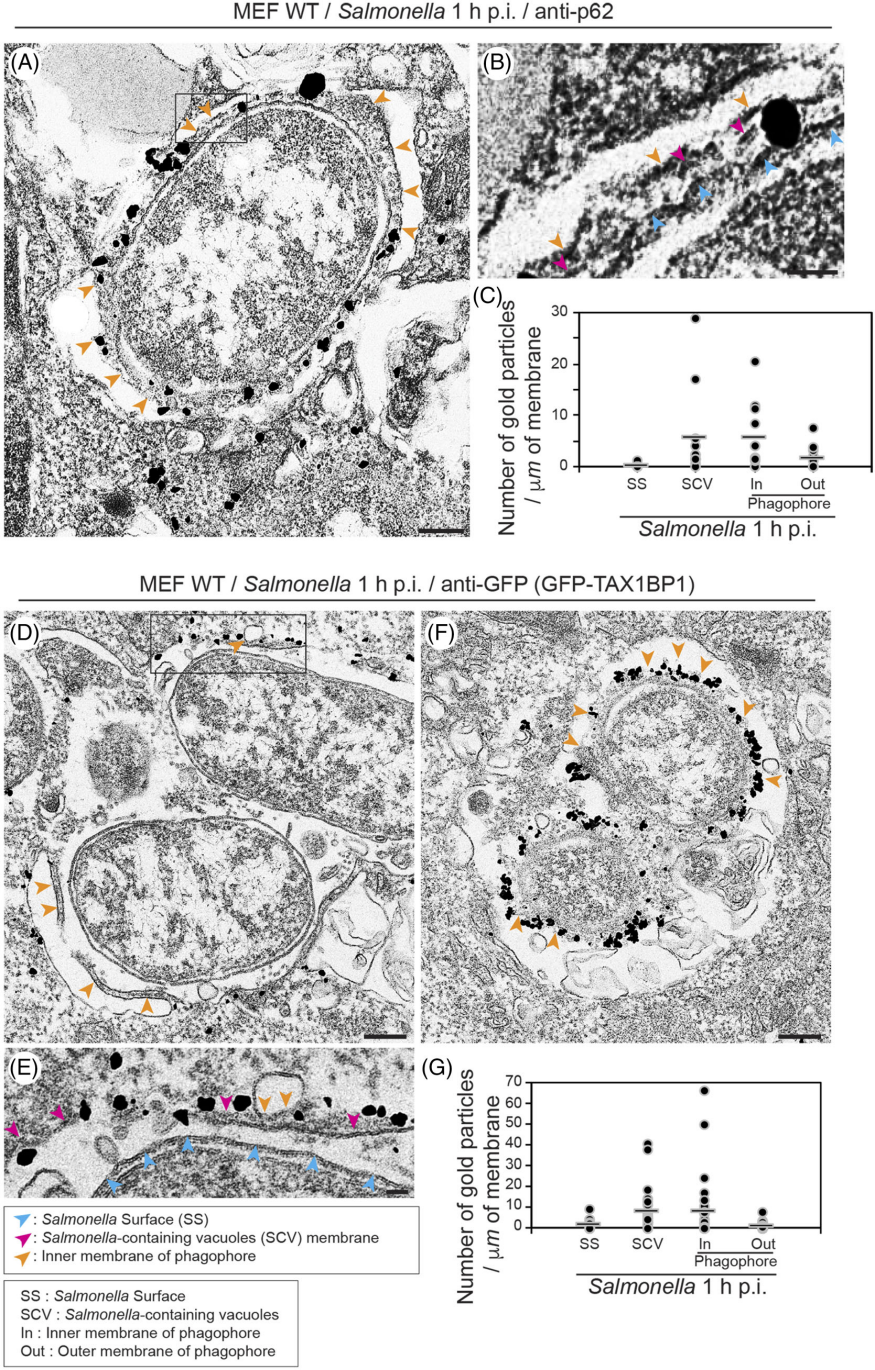
The autophagy receptors, TAX1BP and p62, are present between the *Salmonella* surface and the phagophore. (A) Mouse embryonic fibroblasts (MEF) cells were infected with *Salmonella* and labelled for immunoelectron microscopy with anti‐p62 antibodies. (A) Rectangle shows enlarged area in B. Blue arrowheads indicate the position of the *Salmonella* surface, pink arrowheads *Salmonella*‐containing vacuole (SCV) fragments and orange arrowheads the phagophore membrane. Scale bars, 200 nm (A), 50 nm (B). (C) Quantification of the gold particles of anti‐p62 surrounding *Salmonella*. Bars show average of each column. (D, E and F) MEF cells transiently expressing GFP‐TAX1BP1 were infected for 1 h with *Salmonella*‐Cherry and labelled for immunoelectron microscopy with anti‐GFP antibodies (D, E and F). Rectangle in D is enlarged in E. Orange arrowheads highlight the position of the inner membrane of phagophores. Scale bars, 200 nm (D and F), 50 nm (E). (G) Quantification of the gold particles marking the localisation of GFP‐TAX1BP1 surrounding *Salmonella*. Bars show average of each column

Ubiquitination plays a crucial role in recruiting autophagy receptors, including TAX1BP1 and p62, to pathogens such as *Salmonella*. The autophagy receptors initiate the formation of the phagophore by bridging ubiquitinated substrates and LC3‐membranes through their LC3‐interacting region and their ubiquitin‐binding domain. As the presence of the SCV membrane coincides with the formation of the phagophore (Figures 3, 4 and 5), we next analysed at the ultrastructural level the distribution of the ubiquitin signal and the autophagy receptors. MEFs expressing GFP‐TAX1BP1 were infected for 1 hour with *Salmonella* and pre‐embedding immuno‐EM was performed to detect endogenous p62 or GFP‐tagged TAX1BP1. Overall, both autophagy receptors accumulate on the SCV membrane and in the space between the surface of the pathogen and the phagophore, where remnants of the SCV membrane are often still visible (Figure 6). Our quantitation shows that for both TAX1BP1 and p62, the highest amount of signal is present on the SCV membrane and on the inner membrane of the phagophore but not directly on the *Salmonella* surface (Figure 6C and G). TAX1BP1 is also a cargo adaptor protein for MYO6, a myosin motor that has previously been shown to be recruited to ubiquitin‐positive *Salmonella* that also contain TAX1BP1, p62 as well as LC3.^12^ The ultrastructural analysis shows that MYO6 displays a very similar distribution as TAX1BP1 on the SCV membrane and on the cytosolic outer membrane of the phagophore (Figure S2).

**Figure 7:**
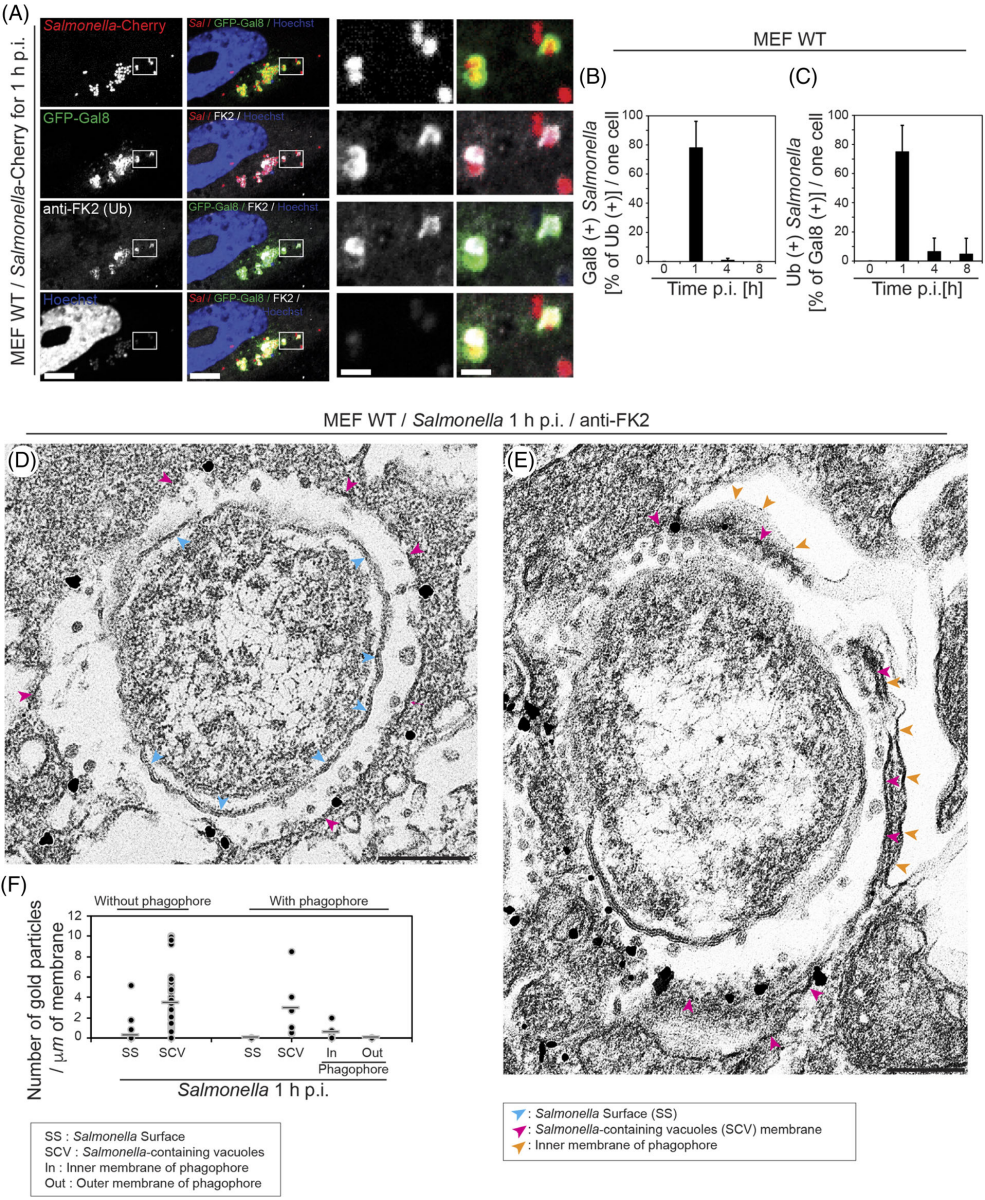
Ubiquitin is present on *Salmonella*‐containing vacuole (SCV) membranes. (A) Mouse embryonic fibroblasts (MEF) cells transiently expressing GFP‐Galectin‐8 were infected with *Salmonella*‐Cherry for 1 h, processed for confocal microscopy and immunostained for Ubiquitin (far red). Nuclei were labelled with Hoechst (blue). Rectangles are shown enlarged in panels on the right side. Scale bars, 10 μm (two rows on the left), 2 μm (two rows on the right). (B) Quantification of the percentage of Galectin‐8‐positive *Salmonellae* that are also ubiquitin‐positive. (C) Quantification of the percentage of ubiquitin‐positive *Salmonellae* that are also Galectin‐8‐positive. (D and E) MEF cells were infected with *Salmonella*‐Cherry for 1 h, processed for immunoelectron microscopy and stained with anti‐FK2 antibodies. Scale bars, 200 nm (D and E). (F) Quantification of the number of gold particles indicating the position of the anti‐ubiquitin antibody. Bars show average of each column

**Figure 8:**
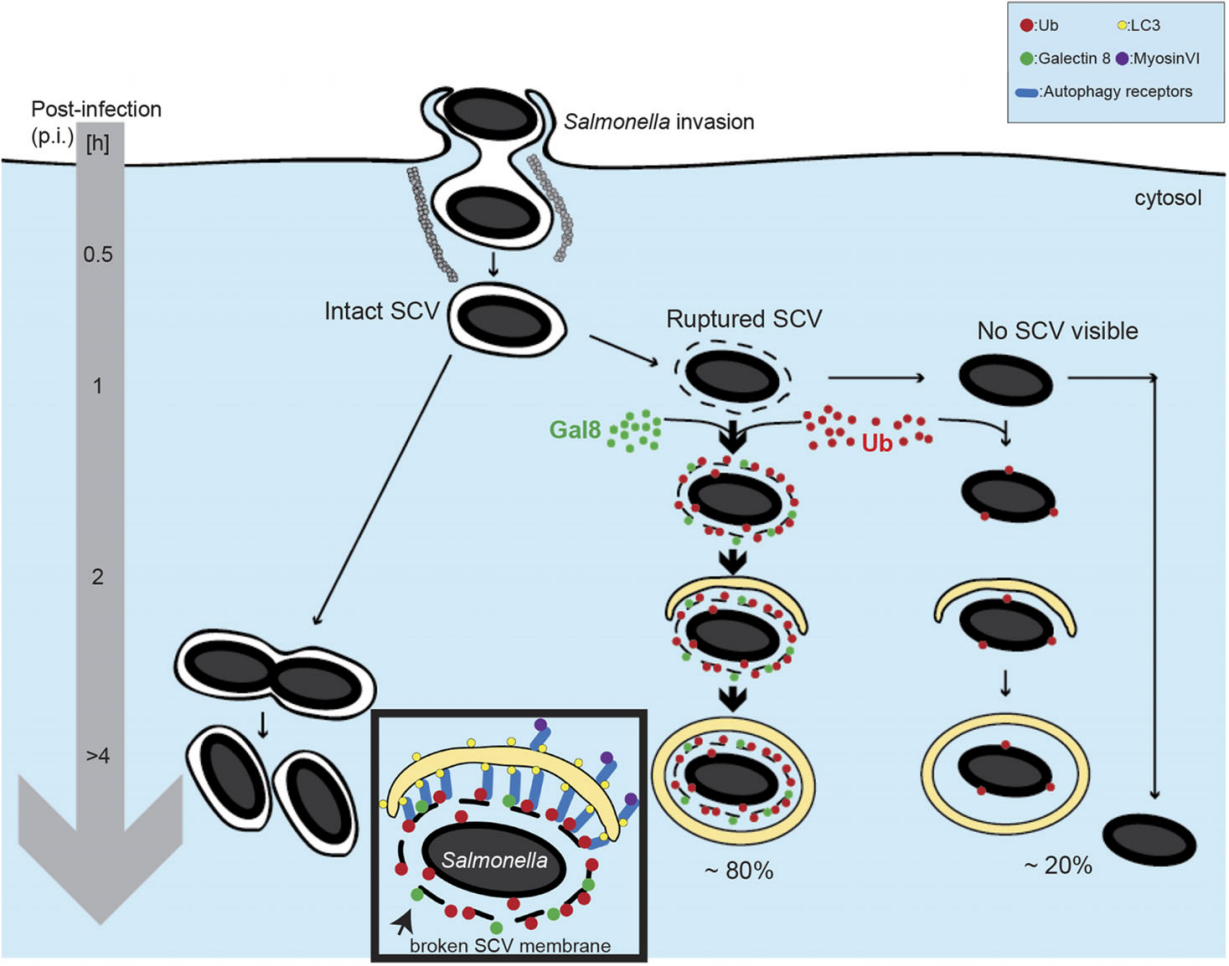
Cartoon illustrating the suggested role of the *Salmonella*‐containing vacuole (SCV) membrane in phagophore formation. Ubiquitin is not only present on the *Salmonella* surface, but also enriched on the fragmented SCV

Next, we visualised the distribution of mono‐ and polyubiquitinylated proteins around Galectin‐8‐positive *Salmonella* using the monoclonal FK2 anti‐ubiquitin antibody, which has been reported to detect K29‐, K48‐ and K63‐linked ubiquitin chains on proteins. MEFs were infected with *Salmonella*‐Cherry and after 1 h processed for immunofluorescence microscopy to determine the number of bacteria that are positive for Galectin‐8 and for ubiquitin, detected with the FK2 antibody (an example is shown in Figure 7A). The results were plotted as the percentage of Galectin‐8‐positive bacteria that also contain ubiquitin (Figure 7B) or the percentage of ubiquitin‐positive bacteria that carry a ubiquitin coat (Figure 7C). Our data show that more than 80% of *Salmonellae* that are positive for Galectin‐8 also contain a ubiquitin signal and more than 70% of *Salmonellae* that are ubiquitin‐positive contain Galectin‐8‐positive SCVs. These results were confirmed by EM, which demonstrates that the majority of the ubiquitin signal is not on the *Salmonella* surface but associated with the SCV irrespective of whether the SCV was already associated with an autophagic phagophore (Figures 7D,E and 8).

## 3 DISCUSSION

The correct deposition of a ubiquitin coat is crucial to create a docking space for the autophagy machinery but also a signalling platform for local nuclear factor kappa B (NFkB) activation. Recent results from a number of labs obtained using super‐resolution microscopy, revealed the uneven distribution of ubiquitin around cytosolic *Salmonella* with different ubiquitin chains segregated into distinct patches.^21^, ^22^ Although these high‐resolution light microscopy approaches allow the visualisation of ubiquitin‐enriched and less dense regions around the bacteria, they do not provide the optical resolution to identify the exact nature and position of the ubiquitin target. We therefore used EM in this study to determine the exact distribution of the ubiquitin coat. Our data clearly demonstrates that the majority of the ubiquitin signal is not present on the outer *Salmonella* membrane, but associated with the membrane of the broken SCV.

Our data are in agreement with previous results from the Randow lab that at early time points (1 h p.i.) about 10% of *Salmonellae* are Galectin‐8 positive (indicating that they have escaped into the cytosol) and also about 10% of *Salmonellae* are positive for FK2‐positive ubiquitin signal.^9^ Therefore, the early response not only involves the Galectin‐8 “eat‐me” signal but also uses ubiquitin as an amplification signal to recruit autophagy receptors leading to phagophore formation around fragments of the SCV. This hypothesis is supported by our finding that “naked” *Salmonella* particles without any SCV fragments detectable at the ultrastructural level, are less frequently surrounded by a phagophore.

Our findings suggest that multiple *Salmonella* particles can exist within one very large phagophore raise some interesting points. It is likely that such membrane nucleation and expansion must depend on tight interactions between selective autophagy adaptors recognising the *Salmonella* eat‐me signals (eg, ubiquitination) and the autophagy machinery, and this probably explains why the phagophore membranes follow tightly the contour of the bacterial outer membrane. Recent work has discovered interactions between NDP52 and the ULK complex as well as between TBK1 and WIPI2.^15^, ^23^ Given the tightness of these engulfing membranes with their targets, additional such interactions will undoubtedly be discovered between adaptors and early autophagy proteins. Of note, the Atg19 cargo receptor in yeast contains multiple Atg8 binding sites, and this facilitates tight wrapping of the autophagic membrane around its cargo.^24^ Similarly, the presence of multiple adaptors on *Salmonella* (including p62, optineurin, TAX1BP1) each with its own LC3 interacting region and the ability to multimerize may create a multivalent interaction with the autophagosomal membrane that would enable tight engulfment. What is less clear is the mechanism by which these large phagophores are formed. In some of our images, we can clearly see what appear to be smaller phagophores fusing together whereas in others there appears to be a continuous phagophore making its way around a cluster of *Salmonella* particles from both directions. The former type of event would predict that there must be some activity within the autophagic components capable of fusing double membrane phagophores, perhaps akin to the final step of phagophore closure. Such activity would also be relevant for cases where a large mitochondrial fragment is enclosed by multiple phagophores fusing together at some point.^25^ The latter type of event (a seemingly continuous phagophore enclosing a very large target) is characterised by autophagosomes beyond the usual size limit that has been reported to range from 0.15 μm diameter for the CVT pathway in yeast to 1 μm diameter for autophagy and mitophagy in mammalian cells.^26^ What mechanisms ensure that the autophagic machinery can stay engaged continuously on structures up to 30 μm in circumference? Are there feedback controls operating for such unusual autophagosomal sizes? If this is in principle possible, why do we also see multi‐phagophore membranes engulfing similarly‐sized *Salmonella* clusters? What determines these two modes of selective autophagy? Answering these questions must be one of the objectives of future work as the precise mechanisms of *Salmonella* autophagy are established.

## 4 MATERIALS AND METHODS

### 4.1 Reagents and antibodies

The following commercial antibodies were used in this study; monoclonal antibodies to GFP (Abcam, ab1218), LC3 (Cosmo Bio, CTB‐LC3‐2‐1C), ubiquitin (clone FK2; Enzo); polyclonal antibodies to *Salmonella* (Abcam), GFP (Life Technologies).

### 4.2 Plasmids

In this study, we used the following plasmids: human TAX1BP1 in pEGFP,^27^ human MYO6 without any splicing inserts in pEGFP,^28^ Galectin‐8 in pEGFP was a kind gift from Felix Randow.^9^

### 4.3 Cell culture

Primary mouse embryonic fibroblasts (MEFs) from wild‐type mice were prepared as previously described^12^ and immortalised using the SV40 large T‐antigen (pEF321‐T). The MEF cells were cultured in Dulbecco's modified Eagle's medium (DMEM) supplemented with 10% foetal bovine serum, 2 mM Glutamine and 100 U/mL penicillin and 100 μg/mL streptomycin under 5% CO_2_ and expanded over several weeks to make mixed immortalised MEF cultures.

### 4.4 Infection of MEFs with *Salmonellla*

*Salmonella enterica* serotype typhimurium (strain 12 023) were a kind gift from F. Randow (MRC LMB, Cambridge, UK). Overnight cultures of *Salmonella* expressing mCherry cDNA were grown at 37°C in Luria Broth (LB) and diluted the next day 1:33 into Luria Broth (LB) containing ampicillin and cultured for a further 3 hours at 37°C. MEF cells were grown for 3 hours at 37°C in serum‐free culture media containing 0.1% Biological Stain Commission (BSA) before infected for 15 minutes with *Salmonella* diluted 1:100 into serum‐free media. After infection, the cells were washed and first grown for 2 hours with complete growth media containing 100 μg/mL Gentamicin before culturing in complete media with 20 μg/mL Gentamicin. For immunofluorescence microscopy experiments, infected cells were fixed in formaldehyde at various times after infection.

### 4.5 Immunofluorescence microscopy

MEFs were plated on glass coverslips, fixed in 4% formaldehyde, permeabilised in 0.02% Triton X‐100 in phosphate‐buffered saline (PBS) and blocked in 1% BSA in PBS. The fixed cells were incubated with primary antibodies at room temperature for 2 hours followed by secondary antibodies conjugated to Alexa fluor 488, 568 or 647. Images were taken on a Zeiss LSM710 confocal microscope with ZeissZEN software. Manual quantitation was performed to determine the % of Galectin‐8‐positive *Salmonella* that were also LC3‐ or ubiquitin‐positive for more than 100 bacteria from multiple cells from three independent experiments.

### 4.6 Electron microscopy

For conventional EM analysis, MEFs were cultured on collagen‐coated plastic coverslips and fixed in mixed solution of 2.5% glutaraldehyde (TAAB, Berkshire, England) and 2.0% formaldehyde (TAAB, Berkshire, England) in 0.1 M sodium phosphate buffer pH 7.4 (phosphate buffer) for 2 h. The cells were washed in the same buffer five times and post‐fixed in 1% osmium tetroxide (Agar) in phosphate buffer for 1 hour and then dehydrated and embedded in Epon 812 (Agar) according to a standard procedure.^29^ Ultrathin sections were stained with uranyl acetate and lead citrate and observed under an FEI Technical Spirit TEM. Images were recorded with a Gatan CCD camera (Gatan US 1000X‐U Camera 2000 kV). For immunoelectron microscopy analysis, cells were fixed with 4% formaldehyde solution (TAAB, Berkshire, England) in phosphate buffer for 2 hours on ice. The pre‐embedding gold enhancement immunogold method was used as described previously.^29^, ^30^

### 4.7 Pre‐embedding immunoEM

Cells were grown on collagen‐coated plastic coverslips and were fixed in 4% formaldehyde (TAAB, Berkshire, England) in 0.1 M phosphate buffer for 1 hour. After washing with 0.1 M phosphate buffer, the cells were dipped in liquid nitrogen to permeabilize the membranes and blocked with 5% BSA (SIGMA‐ALDRICH, A2153), 5% normal goat serum (SIGMA‐ALDRICH, G9023), 0.005% saponin (nacalai tesque, 30 502‐42) and 0.1% cold water fish skin gelatin (SIGMA‐ALDRICH, G7765) in 0.1 M phosphate buffer. The cells were labelled with antibodies against GFP, p62, Ubiquitin, LC3‐positive compartments overnight at 4°C, washed in 0.1 M phosphate buffer containing 0.01% saponin and incubated in goat anti‐mouse IgG conjugated to 1.4‐nm nanogold particles (Nanoprobes, 2002) or anti‐rabbit IgG conjugated to 1.4‐nm nanogold particles (Nanoprobes, 2004). After washing, the gold labelling was intensified by using a Gold Enhance EM kit (Nanoprobes, 2113). After stopping the gold enhancement reaction in 1% aqueous sodium thiosulfate solution, the cells were washed in distilled water and post fixed in 1% OsO4 containing 1.5% K_4_[Fe(CN)_6_] in 0.1 M phosphate buffer for 1 hour. After washing in distilled water, the cells were dehydrated with a graded series of ethanol, infiltrated with resin (Agar Scientific, Agar 100 resin kit, R1031), which was polymerised at 60°C for 2 days. Ultrathin sections were collected onto grids, post stained with uranyl acetate and lead citrate and viewed using an FEI Technical Spirit TEM.

### 4.8 Quantitative analysis of immunogold labelling

Electron micrographs were taken from single ultrathin sections from randomly‐selected fields that included at least one immunolabelling gold particle with an identifiable autophagosome or/and salmonella. Micrographs were analysed with ImageJ‐Win64 software. The Gold particles were counted to be associated with each membrane.

## Supporting information


**Figure S1** *The ultrastructure of* *Salmonella enterica* *serotype Typhimurium (strain 12 023)*. MEF cells were infected with *Salmonella*‐Cherry for 1 hourour before fixation and processing for electron microscopy. (A) Transmission electron microscopy of *Salmonella enterica* serotype typhimurium. (C) MEF cells were labelled for immunoelectron microscopy with anti‐*Salmonella* antibodies. For both panels, arrows highlight the different layers of the *Salmonella* envelope: blue arrow: outer membrane; yellow arrow: periplasm; red arrow: inner membrane. Panels (B and D) represent enlarged boxed regions in (A) and (C). Scale bars, 200 nm (A and C) and 50 nm (B and D).
**Figure S2.** *Myosin VI is present on Salmonella*‐containing vacuole (*SCV*) *and outer membrane of phagophore*. MEF cells transiently expressing GFP‐myosin VI were infected for 2 h with *Salmonella*‐Cherry. Cells were labelled for immunoelectron microscopy with anti‐GFP antibodies. (A and B) Orange arrowheads show inner membrane of the phagophores. Scale bars, 200 nm (a and b). (C) Quantification of the number of gold particles of GFP‐myosin VI surrounding *Salmonella*. Bars show average of each column.
