# Supplementary material for: Ultrastructural insights into pathogen clearance by autophagy
Source: Traffic. 2020 Mar 4;21(4):310–23. doi: 10.1111/tra.12723 (PMC8629118; doi:10.1111/tra.12723)
Supplement: Supplementary file 1 — Figure S1 The ultrastructure of Salmonella enterica serotype Typhimurium (strain 12 023). MEF cells were infected with Salmonella‐Cherry for 1 hourour before fixation and processing for electron microscopy. (A) Transmission electron microscopy of Salmonella enterica serotype typhimurium. (C) MEF cells were labelled for immunoelectron microscopy with anti‐Salmonella antibodies. For both panels, arrows highlight the different layers of the Salmonella envelope: blue arrow: outer membrane; yellow arrow: periplasm; red arrow: inner membrane. Panels (B and D) represent enlarged boxed regions in (A) and (C). Scale bars, 200 nm (A and C) and 50 nm (B and D). Figure S2. Myosin VI is present on Salmonella‐containing vacuole (SCV) and outer membrane of phagophore. MEF cells transiently expressing GFP‐myosin VI were infected for 2 h with Salmonella‐Cherry. Cells were labelled for immunoelectron microscopy with anti‐GFP antibodies. (A and B) Orange arrowheads show inner membrane of the phagophores. Scale bars, 200 nm (a and b). (C) Quantification of the number of gold particles of GFP‐myosin VI surrounding Salmonella. Bars show average of each column. [file TRA-21-310-s001.pdf]

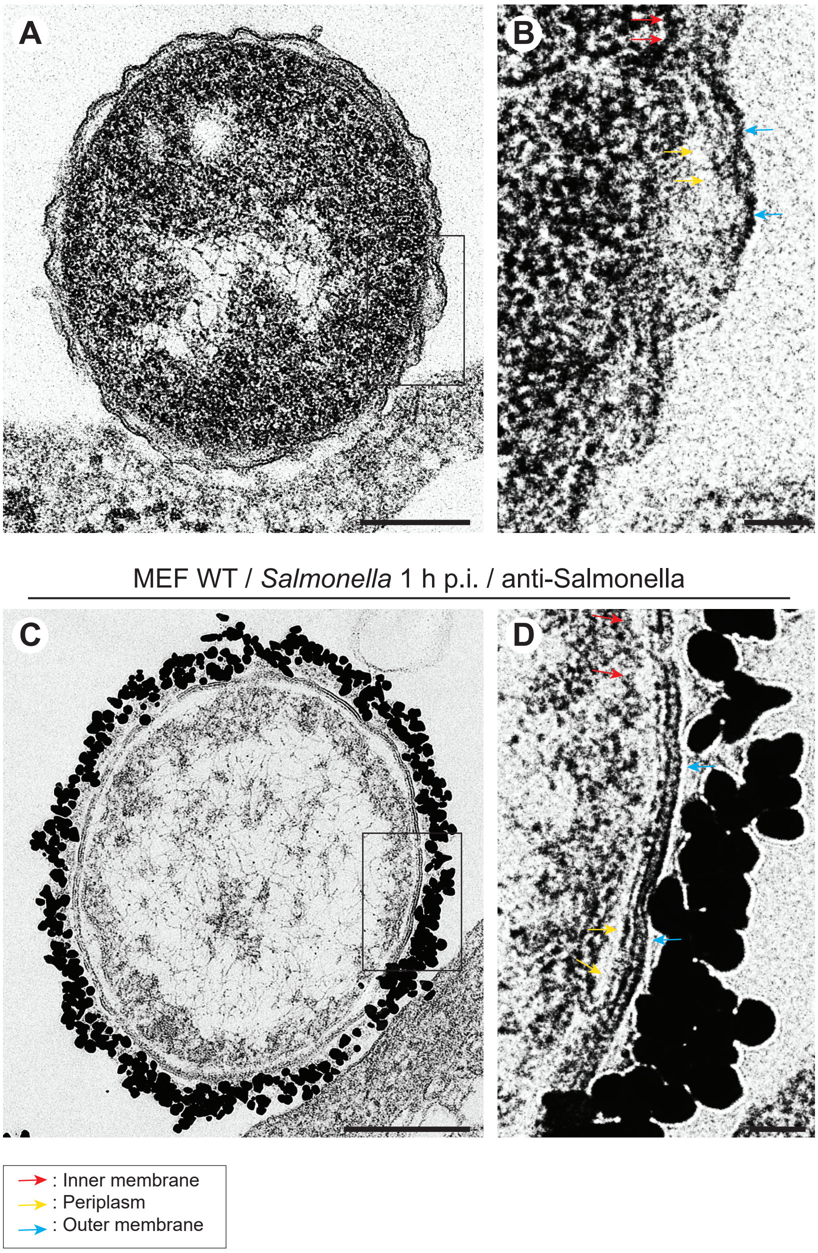

Chieko Kishi-Itakura et al. **Supplementary Figure 1**

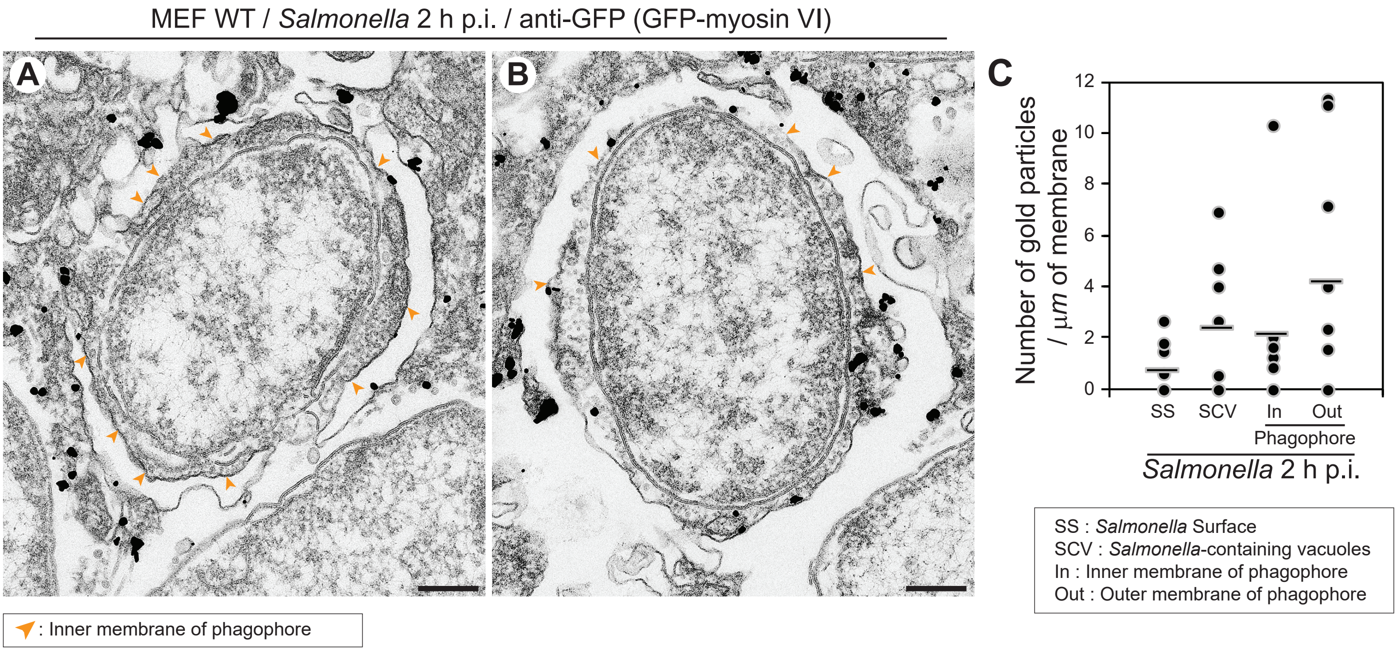

Chieko Kishi-Itakura et al. **Supplementary Figure 2**
